# GSK3 is a negative regulator of the thermogenic program in brown adipocytes

**DOI:** 10.1038/s41598-018-21795-y

**Published:** 2018-02-22

**Authors:** Lasse K. Markussen, Sally Winther, Barton Wicksteed, Jacob B. Hansen

**Affiliations:** 1 Department of Biology, University of Copenhagen, DK-2100 Copenhagen, Denmark; 2 Division of Endocrinology, Diabetes and Metabolism, Department of Medicine, University of Illinois at Chicago, Chicago, IL USA

## Abstract

Brown adipose tissue is a promising therapeutic target in metabolic disorders due to its ability to dissipate energy and improve systemic insulin sensitivity and glucose homeostasis. β-Adrenergic stimulation of brown adipocytes leads to an increase in oxygen consumption and induction of a thermogenic gene program that includes uncoupling protein 1 (*Ucp1*) and fibroblast growth factor 21 (*Fgf21*). In kinase inhibitor screens, we have identified glycogen synthase kinase 3 (GSK3) as a negative regulator of basal and β-adrenergically stimulated *Fgf21* expression in cultured brown adipocytes. In addition, inhibition of GSK3 also caused increased *Ucp1* expression and oxygen consumption. β-Adrenergic stimulation triggered an inhibitory phosphorylation of GSK3 in a protein kinase A (PKA)-dependent manner. Mechanistically, inhibition of GSK3 activated the mitogen activated protein kinase (MAPK) kinase 3/6-p38 MAPK-activating transcription factor 2 signaling module. In summary, our data describe GSK3 as a novel negative regulator of β-adrenergic signaling in brown adipocytes.

## Introduction

The discovery of active brown adipose tissue (BAT) in healthy adults has revitalized the concept of combating metabolic dysfunction via recruitment and activation of brown adipocytes^1–3^. BAT can dissipate energy by uncoupled respiration, a process called adaptive thermogenesis^1–3^. BAT is classically activated by cold, which through sympathetic nervous system-mediated release of norepinephrine at the surface of brown adipocytes activates β-adrenergic receptors. This activation results in augmented lipolysis, mitochondrial uncoupling, oxygen consumption and thermogenesis. In mice, activated BAT promotes glucose and triacylglycerol clearance, improves insulin sensitivity and glucose tolerance, and counteracts obesity^1–3^. Most of these effects of brown adipocytes depend on their high mitochondrial density, the unique presence of uncoupling protein 1 (UCP1) in the inner mitochondrial membrane and a high oxidative capacity^1–3^. In humans, BAT activity correlates with cold-induced energy expenditure and BAT activity is recruited after regular cold exposures^4,5^. In addition, brown-like (also called beige, brite or inducible brown) adipocytes appear in certain white adipose tissues in response to prolonged cold exposure or treatment with β-adrenergic agonists^1–3^.

Recent data suggest that BAT has beneficial metabolic functions beyond thermogenesis which might involve an endocrine role^6,7^. Several signaling molecules with hormonal properties have been found to be released by BAT, particularly under conditions of cold-induced BAT activation^7^. Additionally, the improved glucose tolerance, enhanced insulin sensitivity and decreased adiposity observed with BAT transplantation have also been associated with the endocrine properties of BAT^8,9^. Recent reports have established fibroblast growth factor 21 (FGF21) as a *bona fide* BAT-released factor, secreted by brown adipocytes following cold or β-adrenergic stimulation^10–12^. *Fgf21* expression is controlled by activating transcription factor 2 (ATF2), which in turn is activated by β-adrenergic stimulation in a cAMP-protein kinase A (PKA)-mitogen activated protein kinase (MAPK) kinase 3/6 (MKK3/6)-p38 MAPK-dependent manner^10^. The same intracellular signaling pathway that controls *Fgf21* expression in brown adipocytes is required for induction of a broader thermogenic gene expression program that also includes uncoupling protein 1 (*Ucp1*), type II iodothyronine deiodinase (*Dio2*) and peroxisome proliferator-activated receptor γ co-activator-1α (*Ppargc1α*)^10,13–16^. Pharmacological administration of FGF21 has been ascribed a number of beneficial metabolic effects, including lowering of adiposity and increased glucose tolerance, and some of these effects have been associated with a direct effect of FGF21 on adipocytes^17–19^. Recently, autocrine and/or endocrine actions of FGF21 were shown to induce BAT differentiation and WAT browning in response to activation of G protein-coupled receptor 120^20^. Glycogen synthase kinase 3 (GSK3) is a Ser/Thr kinase implicated in the insulin signaling pathway to control glycogen metabolism, but is now also recognized as a multifunctional kinase regulating an array of additional cellular functions^21^. GSK3 exists as two paralogs: GSK3α and GSK3β. Small-molecule inhibitors of GSK3 have favourable metabolic effects in rodents, including prevention of diet-induced obesity and improved glucose tolerance^22–25^. Thus, GSK3 inhibitors exert some of the same metabolic effects as FGF21 administration.

In a search for novel kinases regulating the thermogenic program of brown adipocytes, we carried out screens with a kinase inhibitor library. We identified GSK3 to be a novel negative regulator of *Fgf21* and thermogenic gene expression in brown adipocytes. Following thermogenic activation, GSK3 becomes inactivated by phosphorylation in a PKA-dependent manner, which in turn leads to increased activity of the MKK3/6-p38 MAPK-ATF2 signaling module. Thus, inhibition of GSK3 unleashes thermogenic signaling in brown adipocytes, an observation pointing to GSK3 as a potentially interesting target in metabolic diseases.

## Results

### FGF21 is under β-adrenergic control in adipose tissue and cultured brown adipocytes

Unbiased kinase inhibitor screens have been applied to successfully identify novel roles for kinases in regulating the formation and function of thermogenic adipocytes^26–28^. Here we aimed to identify novel kinase functions involved in the β-adrenergically induced thermogenic gene program in brown adipocytes. To this end we decided to use *Fgf21* mRNA levels as a read-out due to its reported high inducibility in BAT upon cold exposure and brown adipocytes in response to β-adrenergic stimulation^10^. Before searching for the involvement of novel kinases, we wanted to confirm *Fgf21* as a meaningful read-out in our screens. Gene expression was measured by reverse transcription-quantitative PCR (RT-qPCR).

**Figure 1:**
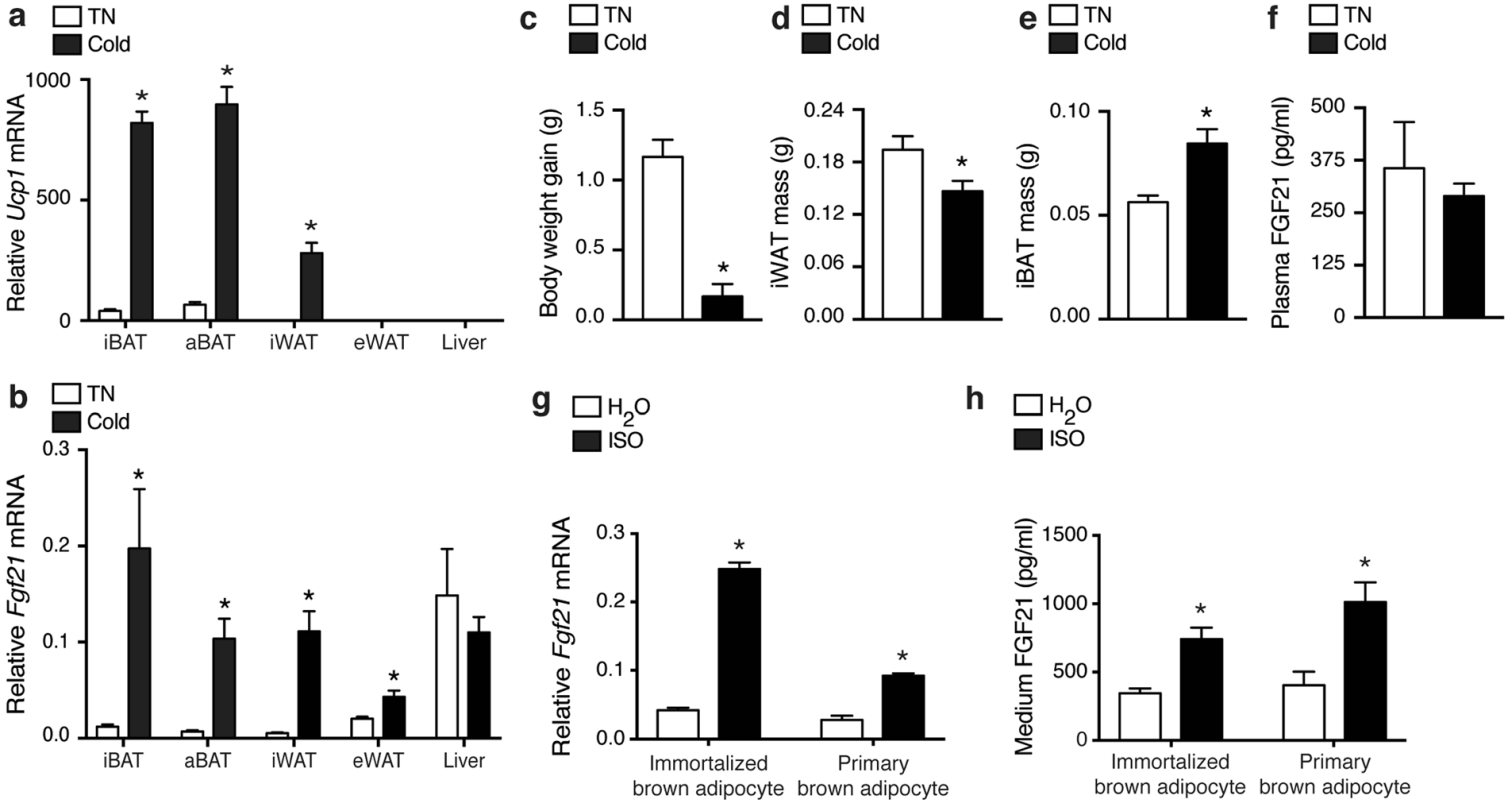
FGF21 expression and secretion is under β-adrenergic control in brown adipocytes. Ten weeks old male C57BL/6JBomTac mice were housed at 29 °C (thermoneutrality, TN) or 4 °C (cold) for 4 days. Relative mRNA levels of *Ucp1* (**a**) and *Fgf21* (**b**) in various tissues. Cold exposure effect on average body weight gain during the 4-day period (**c**), iWAT wet weight (**d**) iBAT wet weight (**e**) and plasma concentration of FGF21 (**f**). (**g**) Expression of *Fgf21* in immortalized or primary brown adipocytes treated with 0.1 μM isoproterenol (ISO) for 6 h. (**h**) Secreted FGF21 in cell culture medium from immortalized or primary brown adipocytes treated with 0.1 μM ISO for 24 h. Data represent mean +SEM (n = 6, mice) or mean of means +SEM (n = 3, cell culture). Unpaired two-tailed Student’s t-test was applied in all panels. *p < 0.05 *versus* TN/H_2_O control.

First, we exposed mice to cold or thermoneutrality for 4 days. Cold exposure led to a significant induction of *Ucp1* mRNA levels in interscapular and axillary BAT (iBAT and aBAT, respectively) and in the browning-prone inguinal WAT (iWAT) (Fig. 1a). *Ucp1* was barely expressed in epididymal WAT (eWAT) and liver, irrespective of housing temperature. In line with previous reports^10–12^, we found that cold exposure strongly increased *Fgf21* expression in thermogenesis-capable adipose tissue depots (BAT and iWAT) and only to a smaller extent in eWAT (Fig. 1b). *Fgf21* expression was high in the liver but did not change with cold exposure. Beside transcriptional changes, cold exposure was associated with a decreased average body weight gain (Fig. 1c), a decreased iWAT mass (Fig. 1d) and an increased iBAT mass (Fig. 1e). However, we did not find the plasma concentration of FGF21 to be significantly altered by the cold exposure (Fig. 1f) despite the enhanced expression in adipose tissues.

Next, we determined *Fgf21* expression in cultured immortalized and primary brown adipocytes in response to β-adrenergic stimulation. In both cell models, *Fgf21* mRNA levels increased after 6 h of stimulation with the pan-β-adrenergic receptor agonist isoproterenol (ISO) (Fig. 1g), which also resulted in a significant increase in medium FGF21 (Fig. 1h).

Thus, in line with previous reports, we show that brown adipocytes induce *Fgf21* expression and secretion following β-adrenergic stimulation in a cell-autonomous manner.

### Kinase inhibitor screen identifies GSK3 as an inhibitor of Fgf21 expression and secretion in brown adipocytes

**Figure 2:**
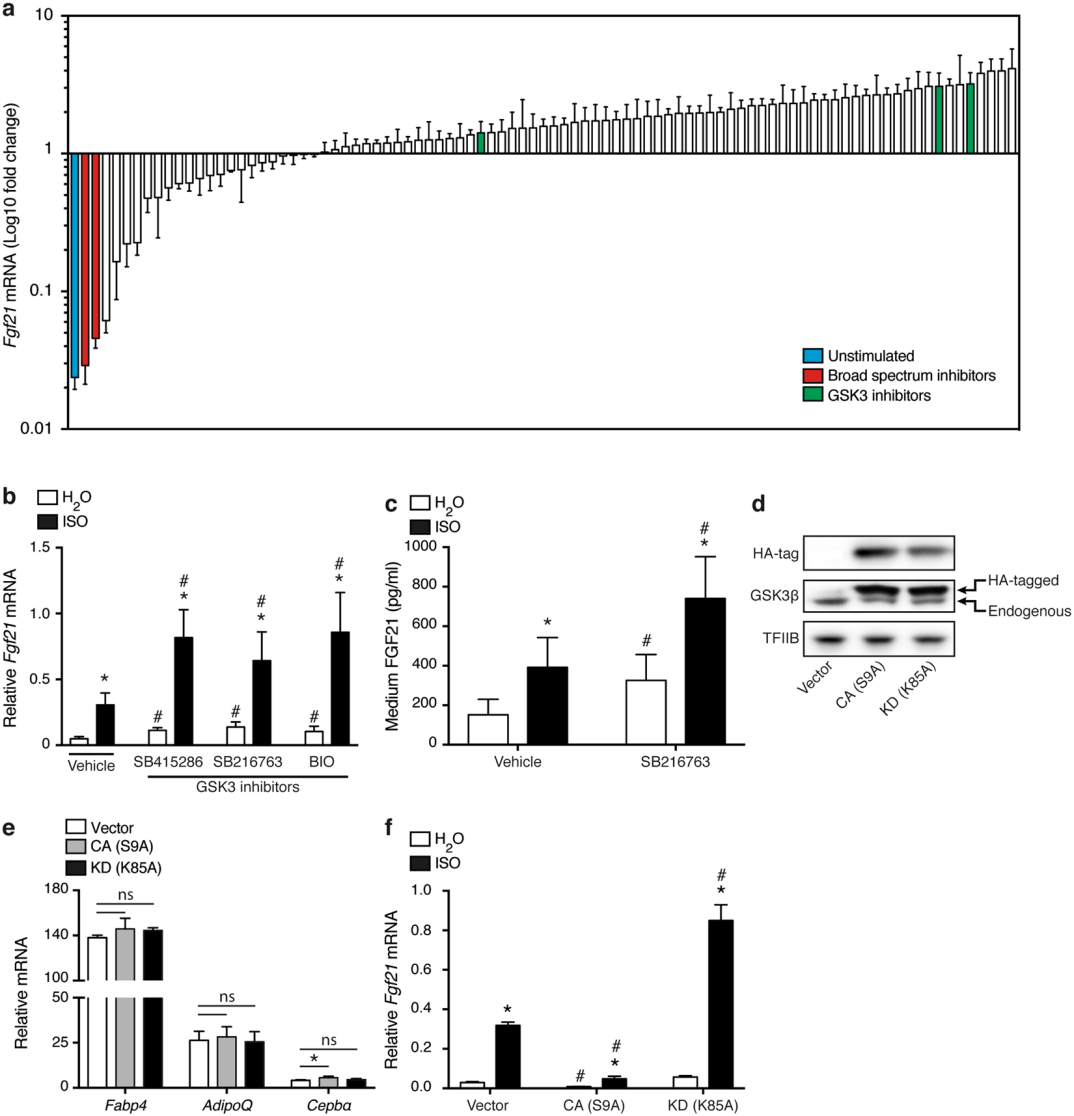
GSK3 is a negative regulator of Fgf21 expression and secretion in brown adipocytes. (**a**) Kinase inhibitor screen to identify novel regulators of *Fgf21* expression in response to β-adrenergic stimulation. *Fgf21* expression in mature immortalized brown adipocytes pre-treated with 10 μM kinase inhibitor for 1 h before stimulation with 0.1 μM ISO for an additional 6 h. Unstimulated (blue) and broad spectrum inhibitor-treated cells (red) serve as controls. Green columns show cells pre-treated with GSK3 inhibitors. (**b**) *Fgf21* mRNA levels in mature immortalized brown adipocytes pre-treated with 10 μM of the GSK3 inhibitors SB415286, SB216763 and BIO for 1 h before being stimulated with 0.1 μM ISO for additional 6 h. (**c**) Secreted FGF21 in cell culture medium from immortalized brown adipocytes pre-treated with 10 μM SB216763 for 1 h before stimulation with 0.1 μM ISO for an additional 24 h. (**d**) Immunoblot against HA-tag and total GSK3β in immortalized brown adipocytes overexpressing constitutively active (CA) or kinase dead (KD) GSK3β or empty vector. TFIIB serves as loading control. Full-length blots/gels are presented in Supplementary Fig. 2. **(e)** Expression of differentiation markers (*Fabp4*, *AdipoQ* and *Cebpα*) in immortalized brown adipocytes after ectopic overexpression of GSK3β mutants. (**f**) *Fgf21* expression in immortalized brown adipocytes overexpressing GSK3β mutants or empty vector. Mature adipocytes were treated with 0.1 μM ISO for 6 h. Data represents mean of means +SEM from 3 independent experiments. Statistical significance was determined by two-way ANOVA with repeated measures and Tukey’s post hoc test for multiple comparisons. *p < 0.05 *versus* H_2_O. ^#^p < 0.05 *versus* vehicle/vector control. No statistical tests were applied to panel a.

To identify novel kinases involved in the β-adrenergically stimulated expression of *Fgf21* in brown adipocytes we carried out a screen with 90 different kinase inhibitors. Mature immortalized brown adipocytes were pre-treated with kinase inhibitor and stimulated with ISO. All samples have been normalized to the ISO-stimulated control cells to depict changes in ISO-induced *Fgf21* mRNA levels (Fig. 2a). The unstimulated control (without ISO; blue column) showed that ISO stimulation increased *Fgf21* expression by ~40 fold. Of notice, the majority of the kinase inhibitors potentiated the effect of ISO. Two broad-spectrum kinase inhibitors (red columns) served as positive controls and were found to largely block ISO-induced *Fgf21* induction. Two GSK3 inhibitors, SB415286 and SB216763, increased the β-adrenergically induced *Fgf21* mRNA expression ~3-fold, placing them among the 10 inhibitors with the strongest potentiating effect. A third GSK3 inhibitor, BIO, elicited a less pronounced potentiating effect on *Fgf21* expression in the screen (~1.4-fold above ISO-stimulation alone). In dedicated GSK3 inhibitor experiments all three inhibitors were found to elevate both basal and ISO-induced expression of *Fgf21* expression by ~2 fold (Fig. 2b). In these experiments, BIO was as potent as SB415286 and SB216763. Treatment with SB216763 also increased both basal and ISO-induced FGF21 secretion into the cell culture medium (Fig. 2c). In addition to BAT, other key metabolic tissues such as liver, WAT and skeletal muscle also express *Fgf21*^19^. Interestingly, SB216763 only increased basal expression of *Fgf21* in mouse and human white adipocyte cell models (3T3-L1 and hMADS), but not in different hepatocyte cell models or muscle cell lines (Supplementary Fig. 1), suggesting that GSK3 might be an adipocyte-selective regulator of FGF21.

To confirm the kinase inhibitor results by genetic means, retroviral vectors encoding constitutively active (S9A) or kinase dead (K85A) HA-tagged human GSK3β were expressed in immortalized brown pre-adipocytes (Fig. 2d). Overexpression of GSK3β mutants did not appear to affect differentiation as indicated by normal expression of adipose-specific markers fatty acid binding protein 4 (*Fabp4)*, adiponectin (*AdipoQ*) and CCAAT/enhancer binding protein α (*Cebpα)* (Fig. 2e). Constitutively active GSK3β decreased both basal and ISO-induced *Fgf21* expression. The kinase dead mutant had a modest non-significant effect on the basal expression, but increased ISO-induced *Fgf21* expression ~2–3 fold (Fig. 2f). Taken together, we have identified GSK3 as an adipocyte-selective negative regulator of FGF21.

### GSK3 is a negative regulator of the thermogenic gene program

**Figure 3:**
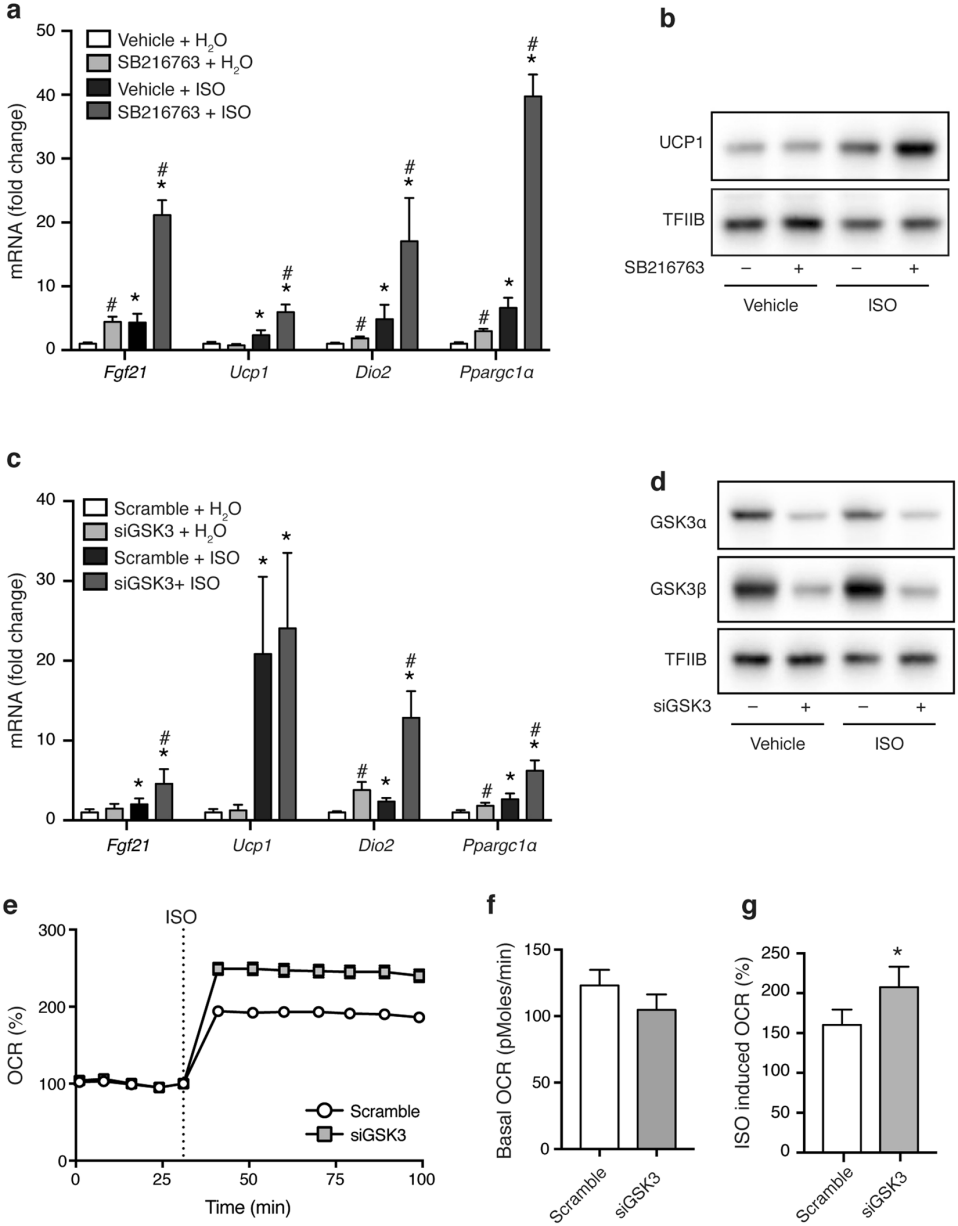
GSK3 restricts the thermogenic program in brown adipocytes. (**a**) Expression of thermogenic genes (*Fgf21*, *Ucp1*, *Dio2* and *Ppargc1α*) in primary brown adipocytes pre-treated with 10 μM SB216763 for 1 h before being stimulated with 0.1 μM ISO for an additional 6 h. (**b**) Immunoblot analysis of UCP1 in primary brown adipocytes pre-treated with 10 μM SB216763 for 1 h before being stimulated with 0.1 μM ISO for an additional 24 h. TFIIB serves as loading control. (**c**) Expression of thermogenic genes (*Fgf21*, *Ucp1*, *Dio2* and *Ppargc1α*) in siRNA-transfected primary brown adipocytes stimulated with 0.1 μM ISO for 6 h. (**d**) Immunoblot analysis of GSK3α and GSK3β in siRNA-transfected primary brown adipocytes stimulated with 0.1 μM ISO for 24 h. TFIIB serves as loading control. (**e**) Representative normalized Seahorse run of oxygen consumption rates (OCR) in siRNA-transfected primary brown adipocytes. Quantification of basal (**f**) and ISO (1 μM)-induced (**g**) OCR. RT-qPCR data are presented as mean of means (+SEM) (n = 4). Statistical significance was determined by two-way ANOVA with repeated measures and Tukey’s post hoc test for multiple comparisons. *p < 0.05 *versus* H_2_O. ^#^p < 0.05 *versus* vehicle/scramble. Seahorse data are presented as mean (±SEM) of one representative experiment (**e**) or 3 independent experiments **(f,g**) and significance was determined by paired t-test (*p < 0.05). For panels b and d, full-length blots/gels are presented in Supplementary Fig. 2.

Next, we wanted to investigate if the effect of GSK3 was specific to the *Fgf21* gene, or if GSK3 might regulate a broader thermogenic gene program in brown adipocytes. Therefore, we measured the effect of SB216763 on expression of the β-adrenergically induced thermogenic genes *Ucp1*, *Dio2* and *Ppargc1α*, in addition to *Fgf21*, in primary brown adipocytes. In these cells, SB216763 augmented ISO-induced expression of *Fgf21*, *Ucp1*, *Dio2* and *Ppargc1α* (Fig. 3a). Basal expression was also increased by SB216763 for *Fgf21*, *Dio2* and *Ppargc1α*, but not for *Ucp1*. The increased *Ucp1* mRNA levels translated into an increase in ISO-induced UCP1 protein (Fig. 3b). Consistent with the effects of SB216763, simultaneous siRNA-mediated knockdown of both GSK3 paralogs in mature primary brown adipocytes significantly induced the expression of *Dio2* and *Ppargc1α* at both the basal and β-adrenergically induced level (Fig. 3c). Knockdown of GSK3 caused an increased expression of *Fgf21* in response to ISO stimulation, but did not influence basal *Fgf21* expression. There was no effect of GSK3 knockdown on expression of *Ucp1* (Fig. 3c) even though GSK3α and GSK3β protein levels were both substantially reduced by the reverse GSK3 siRNA transfection (Fig. 3d).

The effect on the broader thermogenic gene program prompted us to measure the impact of GSK3 on oxygen consumption. Knockdown of both GSK3 paralogs in mature primary brown adipocytes caused a ~30% higher ISO-induced oxygen consumption rate compared with control cells (Fig. 3e,g), while no significant effect was observed under basal conditions (Fig. 3f).

Overall, this indicates that reduction of GSK3 activity in primary brown adipocytes activates the thermogenic gene program and oxygen consumption in response to β-adrenergic stimulation, consistent with GSK3 being a negative regulator of brown adipocyte thermogenic function.

### GSK3 is inactivated after cold exposure and β-adrenergic stimulation in a PKA-dependent manner

**Figure 4:**
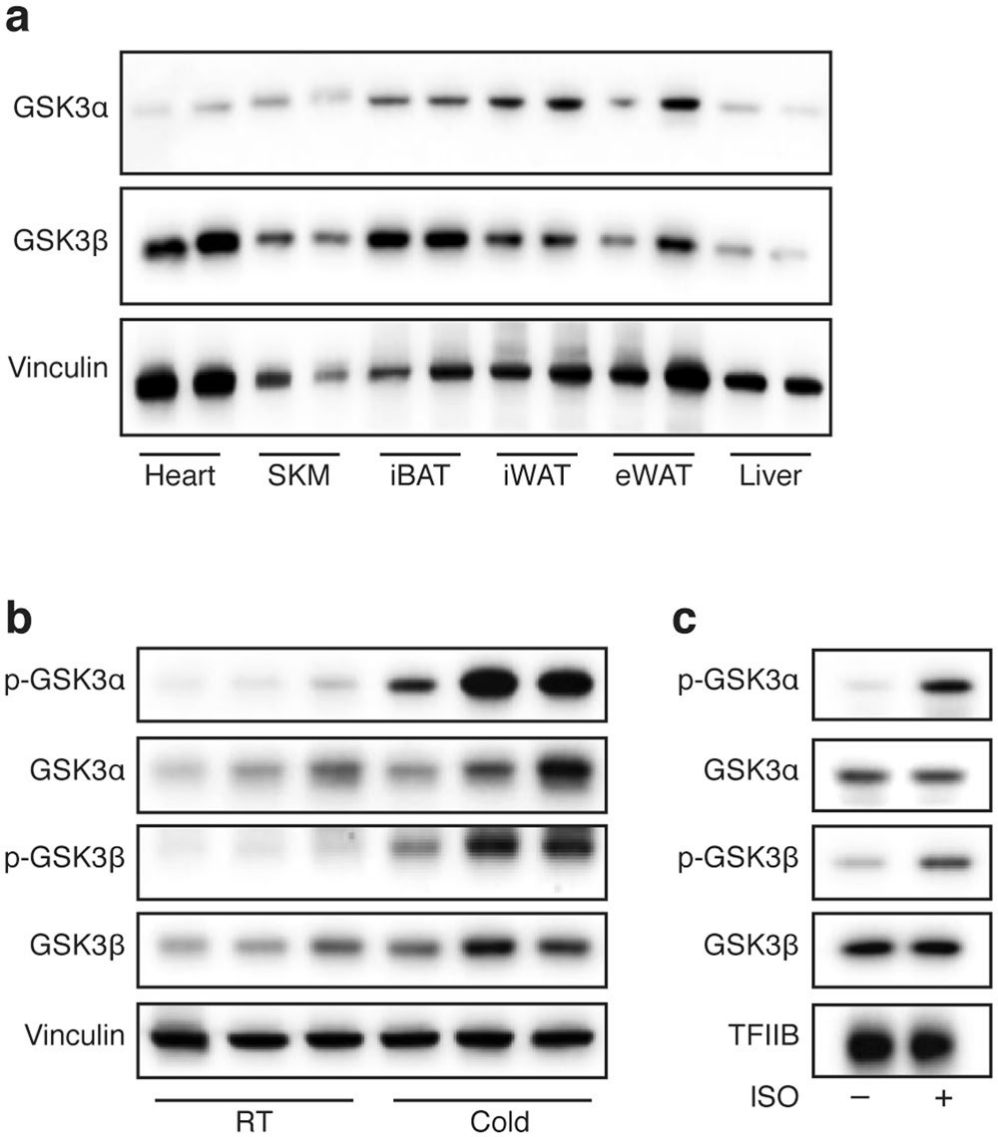
Cold and β-adrenergic signaling cause inhibitory phosphorylation of GSK3 in brown adipocytes. (**a**) Immunoblot analysis of total GSK3α and GSK3β in various key metabolic tissues from 10 weeks old male C57BL/6JBomTac mice housed at room temperature; SKM (skeletal muscle). (**b**) Immunoblot analysis of total and phosphorylated GSK3α and GSK3β in iBAT from 10 weeks old male C57BL/6JBomTac mice housed at room temperature (RT) or 4 °C (cold) for 24 h. (**c**) Immunoblot analysis of total and phosphorylated GSK3α and GSK3β in immortalized brown adipocytes treated with 0.1 μM ISO for 1 h. Vinculin (tissues) or TFIIB (cells) serves as loading control. Full-length blots/gels are presented in Supplementary Fig. 2.

GSK3 is believed to be ubiquitously expressed^29^. Probing various metabolically active tissues such as BAT, WAT, liver, skeletal muscle and heart confirmed expression of both paralogs in all tissues, albeit at varying levels (Fig. 4a). GSK3 is thought to be constitutively active in most tissues under normal physiological conditions, and its activity is mainly regulated by post-translational phosphorylation of an inhibitory amino acid residue: Ser21 in GSK3α and Ser9 in GSK3β^21^. Interestingly, iBAT from mice exposed to cold for 24 h showed a marked increase in the inhibitory phosphorylation of both GSK3α and GSK3β compared to mice housed at ambient temperature (Fig. 4b). This observation was mimicked in immortalized brown adipocytes, as ISO stimulation increased the inhibitory phosphorylation of both GSK3 paralogs (Fig. 4c). Thus, GSK3 becomes inactivated by phosphorylation in brown adipocytes in response to β-adrenergic stimulation.

**Figure 5:**
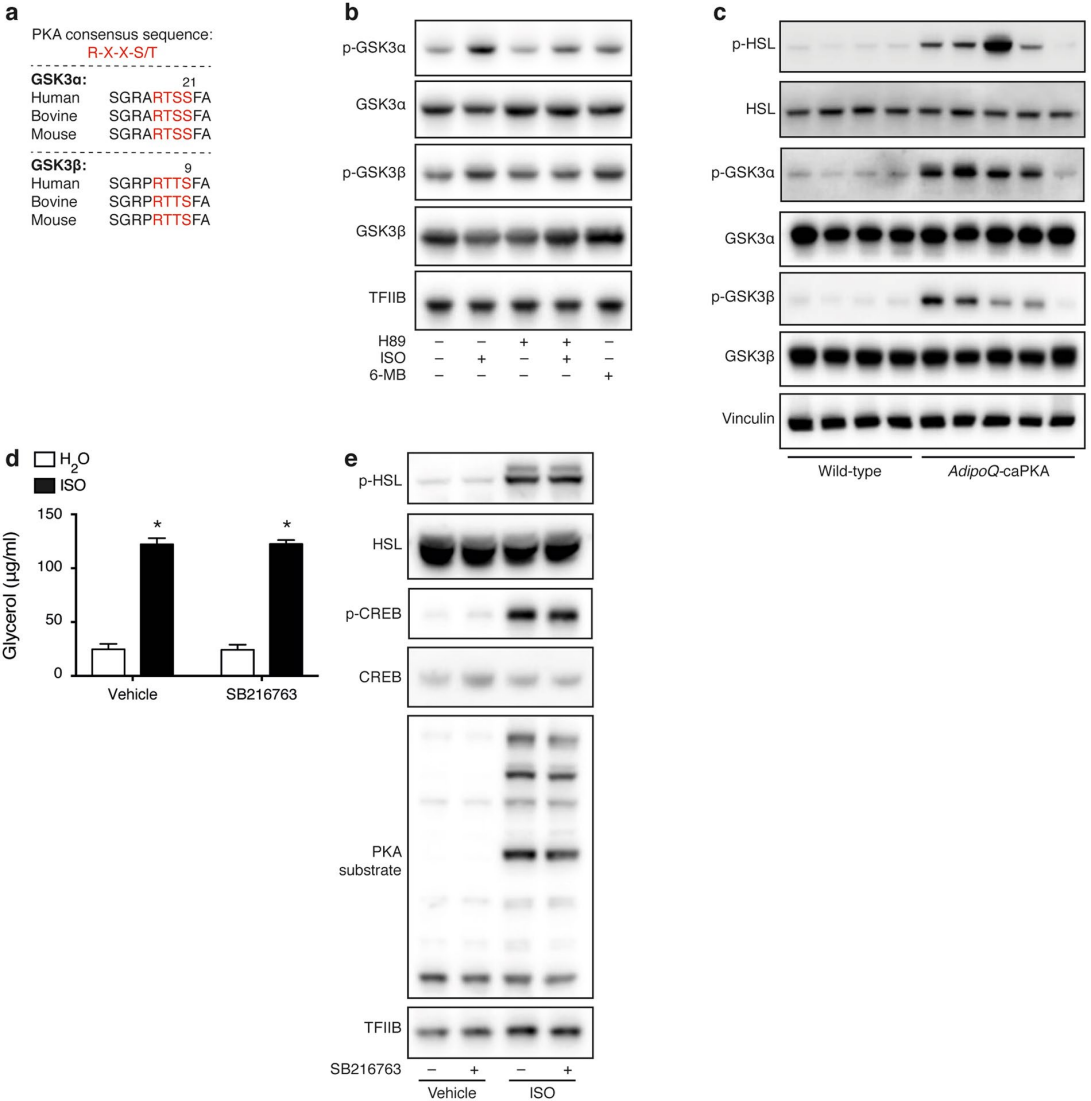
β-Adrenergic GSK3 inactivation is PKA dependent. (**a**) The PKA consensus sites around Ser21 and Ser9 in GSK3α and GSK3β, respectively, are conserved in various species. (**b**) Immunoblot analysis of total and phosphorylated GSK3α and GSK3β in immortalized brown adipocytes treated with 40 μM H89 for 1 h before stimulation with 0.1 μM ISO for additional 15 min. Some of the cells were stimulated with 100 μM 6-MB-cAMP (6-MB) for 15 min. (**c**) Immunoblot analysis of total and phosphorylated GSK3α and GSK3β as well as phosphorylated HSL (Ser660) in iBAT from *AdipoQ*-caPKA and wild-type mice housed at room temperature. (**d**) Medium glycerol of immortalized brown adipocytes pre-treated with 10 μM SB216763 for 1 h before stimulation with 0.1 μM for 24 h. (**e**) Immunoblot analysis of phosphorylated and total HSL (Ser660), CREB (Ser133) and phosphorylated PKA substrates in immortalized brown adipocytes pre-treated with 10 μM SB216763 for 1 h before stimulation with 0.1 μM ISO for 1 h. Vinculin (tissues) or TFIIB (cells) serves as loading control. Data presented as mean of means +SEM (n = 4). Statistical significance was determined by two-way ANOVA with repeated measures and Tukey’s post hoc test for multiple comparisons. *p < 0.05 *versus* H_2_O, comparison between vehicle and SB216763 not significant (2d). For panels b, c and e, full-length blots/gels are presented in Supplementary Fig. 2.

The major transducer of the β-adrenergic signal in brown adipocytes is PKA^30^ and the amino acid sequence around Ser21 in GSK3α and Ser9 in GSK3β is a high probability PKA consensus phosphorylation sequence (Fig. 5a). In addition, GSK3 activity can be inhibited by PKA through phosphorylation of these serine residues in HEK293 and NIH3T3 cells^31^. Pre-treatment of immortalized brown adipocytes with the PKA inhibitor H89 before β-adrenergic stimulation blunted the ISO-induced phosphorylation of both GSKα and GSK3β (Fig. 5b). Stimulation with the PKA-specific activator 6-MB-cAMP mimicked the effect of ISO on Ser9-phosphorylation of GSK3β, and to a smaller extent on Ser21-phosphorylation of GSK3α. iBAT from mice expressing a constitutively active mutant allele of the PKA catalytic subunit α specifically in adipose tissue (*AdipoQ*-caPKA mice)^32,33^ revealed a hyperphosphorylated pattern of the PKA substrate hormone sensitive lipase (HSL) as well as of GSK3α and GSK3β compared with control mice (Fig. 5c). Treatment of immortalized brown adipocytes with SB216763 did not result in increased basal or ISO-induced lipolysis (Fig. 5d) or altered phosphorylation status of major PKA target proteins (HSL, cAMP responsive element-binding protein (CREB) and PKA substrates detected with an anti-PKA substrate antibody) (Fig. 5e), suggesting that GSK3 does not interfere with PKA activity, but rather acts downstream of PKA in the β-adrenergic signaling pathway.

In summary, these data suggest that the inactivating phosphorylation of GSK3 in response to β-adrenergic stimulation is dependent on PKA activity in brown adipocytes.

### Inhibition of GSK3 promotes activation of the MKK3/6-p38 MAPK-ATF2 signaling module

**Figure 6:**
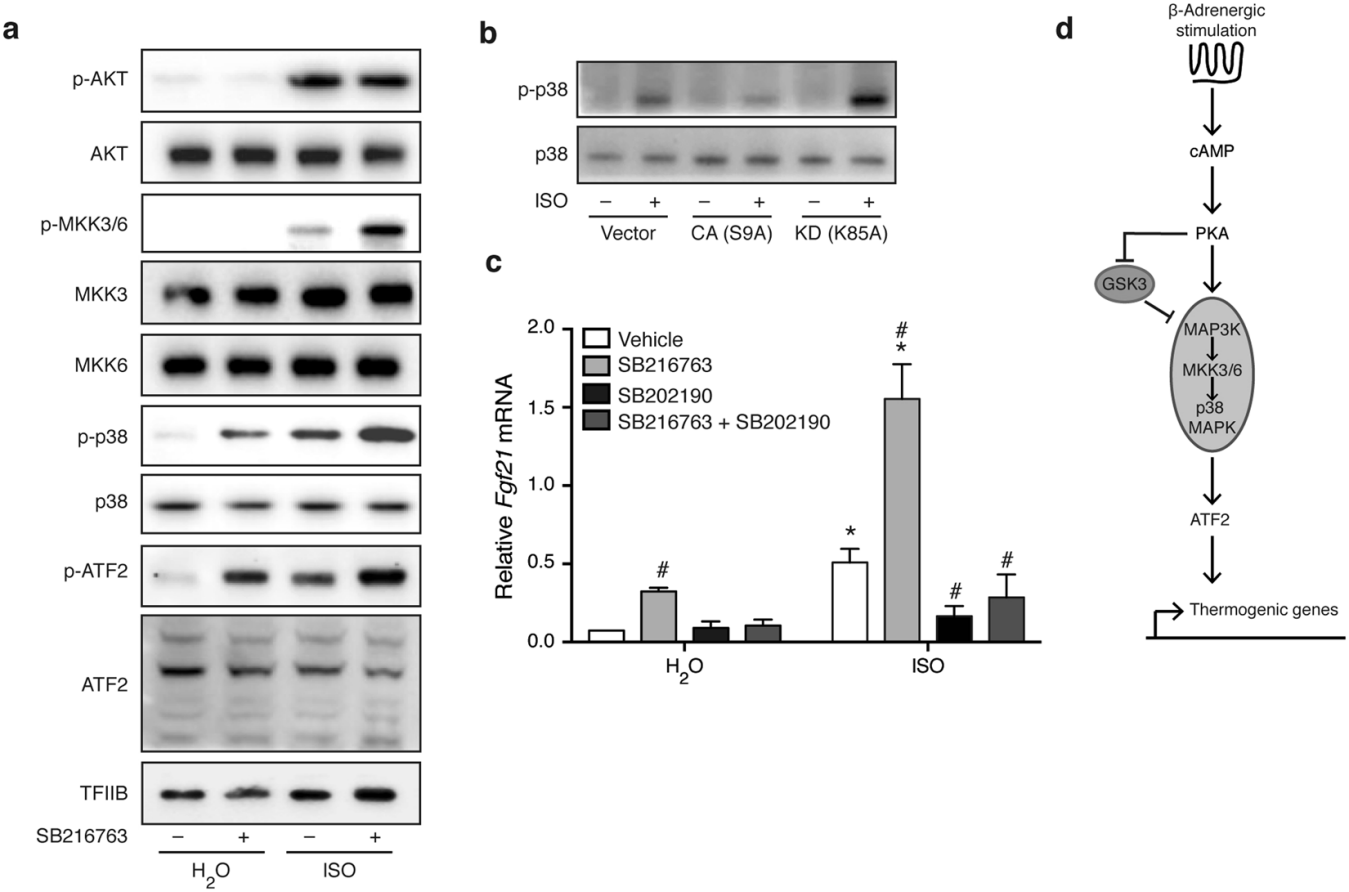
Inhibition of GSK3 promotes activation of the MKK3/6-p38 MAPK-ATF2 signaling module. (**a**) Immunoblot analysis for activating phosphorylations of proteins of the MKK3/6-p38 MAPK-ATF2 signaling module in primary brown adipocytes pre-treated with 10 μM SB216763 for 1 h before stimulation with 0.1 μM ISO for 1 h. (**b**) Immunoblot analysis of total and phosphorylated p38 MAPK in immortalized brown adipocytes overexpressing GSK3β mutants or empty vector. Cells were treated with 0.1 μM ISO for 1 h. (**c**) *Fgf21* expression in primary brown adipocytes pre-treated with 10 μM SB202190 (p38 MAPK inhibitor) for 1 h before treatment with 10 μM SB216763 (GSK3 inhibitor) for 1 h, followed by stimulation with 0.1 μM ISO for additional 6 h. (**d**) Schematic presentation of the proposed mechanism through which GSK3 regulates the thermogenic gene program in brown adipocytes. Data presented as mean of means +SEM (n = 3). Statistical significance was determined by two-way ANOVA with repeated measures and Tukey’s post hoc test for multiple comparisons. *p < 0.05 *versus* H_2_O. ^#^p < 0.05 *versus* vehicle. For panels a and b, full-length blots/gels are presented in Supplementary Fig. 2.

Activation of the thermogenic gene program has been shown to depend on the MKK3/6-p38 MAPK-ATF2 signaling module that is activated downstream of PKA^10,13–15^. Treatment with SB216763 led to increased activating phosphorylations of MKK3/6, p38 MAPK and ATF2 in response to ISO stimulation (Fig. 6a). Moreover, SB216763 augmented the phosphorylation of p38 MAPK and ATF2 even in the absence of β-adrenergic stimulation. These observations suggest that GSK3 activity normally inhibits this signaling module (Fig. 6a). Forced expression of a kinase dead version of GSK3β caused increased phosphorylation p38 MAPK in response to ISO, whereas a constitutively active GSK3β mutant blunted the normal ISO-induced p38 MAPK phosphorylation (Fig. 6b). Furthermore, the effect of SB216763 on both basal and β-adrenergically induced expression of *Fgf21* was abolished in cells pre-treated with the p38 MAPK inhibitor SB202190 (Fig. 6c). As expected, inhibition of p38 MAPK alone attenuated ISO-induced *Fgf21* expression. Thus, these data demonstrate that GSK3 restricts p38 MAPK signaling in brown adipocytes and that the effect of GSK3 inhibition depends on p38 MAPK activity. A schematic model of the proposed novel regulatory circuit of GSK3 inhibition of the MKK3/6-p38 MAPK-ATF2 module in brown adipocytes is illustrated in Fig. 6d.

## Discussion

In energy metabolism research, much effort is put into elucidating how to increase catabolic pathways, such as β-adrenergically induced thermogenesis in brown adipocytes. Here we show that under basal conditions, the expression of thermogenic genes in brown adipocytes is repressed through a GSK3-dependent inhibitory effect on the MKK3/6-p38 MAPK-ATF2 signaling module. Upon β-adrenergic stimulation, brown adipocytes partly release this repression on the thermogenic gene program through a PKA-dependent inactivation of GSK3 to expand the thermogenic transcriptional machinery, thereby forming a novel regulatory circuit.

GSK3 is known to be involved in glycogen storage and insulin signaling, but is now regarded as a multifunctional kinase regulating an array of cellular functions^21^. In kinase inhibitor screens, we identified GSK3 as a negative regulator of the β-adrenergically stimulated increase in *Fgf21* expression in mouse brown adipocytes (Fig. 2a). We confirmed the involvement of GSK3 by various approaches: chemical inhibitors, retroviral overexpression of mutant proteins, and siRNA-mediated knockdown. Inhibition of GSK3 by chemical inhibitors or depletion of GSK3 by siRNA was carried out in the mature adipocyte state, whereas retroviral transduction was performed in preadipocytes. Even though GSK3 activity has been associated with adipogenesis in different adipocyte cell models (3T3-L1 and hMADS)^34–36^, we observed no effect of the GSK3β mutants on adipocyte differentiation (Fig. 2e). This might be due to redundant effects of the two paralogs, where GSK3α was possibly able to sustain normal differentiation. Since forced expression of GSK3β mutants was sufficient to cause effects on *Fgf21* expression, it is possible that the GSK3 paralogs do not exert redundant effects on controlling *Fgf21* levels. Non-redundant effects of the paralogs have been described, e.g. the GSK3β-regulated control of mitochondrial elongation induced by PKA signaling^37^. The catalytic domains of GSK3α and GSK3β are very similar but they differ in their C- and N-terminal regions^21^. However, from our results, we are unable to conclude on the relative contribution of each GSK3 paralog.

When analysing other thermogenic genes, we found that GSK3 was not just a regulator of *Fgf21* expression, but had a broader effect on β-adrenergically stimulated gene transcription (Fig. 6a,b). Similar to *Fgf21*, *Dio2* and *Ppargc1α* expression was increased with GSK3 inhibition or knockdown, both basally and following stimulation. *Ucp1* expression was augmented following β-adrenergic stimulation in GSK3 inhibitor-treated cells, which also correlated with increased protein levels, however basal levels were not affected. We achieved an efficient siRNA-mediated GSK3 knockdown (Fig. 6d), and even though the effects of SB216763 on *Fgf21*, *Dio2* and *Ppargc1α* were mimicked by the knockdown, the effect on *Ucp1* was not. In addition, we observed that knockdown of GSK3 led to increased oxygen consumption following β-adrenergic stimulation (Fig. 3e,g), which might be a consequence of increased levels of *Ppargc1a* and *Dio2* (Fig. 3c) and thereby mitochondrial biogenesis and intracellular conversion of T_4_ to T_3_.

Because of its effect on a range of thermogenic genes, we hypothesised that GSK3 activity would impact a common regulatory pathway such as the MKK3/6-p38 MAPK-ATF2 module, which is well described as controlling *Fgf21*, *Ucp1* and *Ppargc1α* expression in brown adipocytes^10,13–15^. We found that inhibition of GSK3 promoted activation of MKK3/6, p38 MAPK and ATF2, and that functional p38 MAPK signaling was necessary for the observed effects of a GSK3 inhibitor (Fig. 6). Therefore, our data suggest that GSK3 suppresses the signal upstream of MKK3/6. MKK3/6 is activated by members of the MAPK kinase kinase (MAP3K) family^38^. MAP3K involvement in controlling brown adipocyte gene expression is less well understood, however a few of the family members have been linked to specific functions in brown adipocytes. Recently, it was discovered that MAP3K5 regulates brown and beige adipocyte function^39^ and a second MAP3K, MAP3K4, has previously been reported to enhance thermogenic gene expression in adipocytes through activated growth arrest and DNA-damage-inducible 45γ in a PKA-dependent manner^40^. Interestingly, MAP3K4 contains several GSK3 consensus phosphorylation sites and GSK3 has been identified as a negative regulator of MAP3K4 through binding to its kinase domain in COS cells and primary embryonic fibroblasts^41^. It is thus possible that GSK3 impacts the MKK3/6-p38 MAPK-ATF2 module by negatively interfering with the activity of a specific MAP3K, such as MAP3K4, thereby restricting the entire signaling module.

Interestingly, only few negative regulators of the adipocyte thermogenic gene program have been identified, and most of these work by targeting PGC-1α stability or thermogenic genes directly by binding to promoter regions^42^. Here we have identified GSK3 as a novel negative regulator of the brown adipocyte thermogenic gene program, through active suppression of the PKA-dependent MKK3/6-p38 MAPK-ATF2 signaling module (Fig. 6d). For the upstream regulation of GSK3 activity, we show that following cold and β-adrenergic stimulation, the thermogenic gene program in brown adipocytes is de-repressed through induction of inhibitory phosphorylations of GSK3 (Fig. 4b,c). Recently, similar findings on GSK3 phosphorylation were made in 3T3-L1 cells after stimulation with ISO^43^. Moreover, two independent *in vitro* studies and one *in vivo* study, all in cardiomyocytes, have shown that GSK3α and GSK3β become inactivated when treated with ISO^44–46^. Since PKA is the master regulator of brown adipocyte activity and thermogenic gene expression following cold or β-adrenergic stimulation^30^, we speculated that GSK3 inactivation might be downstream of PKA. Indeed, GSK3 inactivation was PKA-dependent *in vitro* and *in vivo* (Fig. 5b,c). However, one *AdipoQ*-caPKA mouse did not appear to exhibit increased PKA activity, as evident by lack of phosphorylation of HSL, and consistently displayed lower levels of phosphorylated GSK3 compared to the other *AdipoQ*-caPKA mice (Fig. 5c**)**. In line with our results, it has previously been shown that PKA, upon stimulation of NIH-3T3 and HEK293 cells with ISO, can inactive GSK3α and GSK3β by phosphorylation at Ser21 and Ser9, respectively^31^. Similarly, it was recently shown that the GSK3 interaction protein, GSKIP, facilitates the phosphorylation of GSK3 by PKA at Ser9 and Ser21 and thereby its inhibition in HEK293 cells^47^. Therefore, it seems likely that GSK3 inactivation following ISO stimulation of brown adipocytes is also a direct consequence of PKA phosphorylation. However, we cannot rule out that other GSK3 kinases are responsible for the phosphorylation of Ser9 and Ser21. One such candidate kinase is AKT that is itself regulated by β-adrenergic receptor activation (Fig. 6a). Beside the inhibitory phosphorylation at Ser9/Ser21, an inhibitory phosphorylation at Ser389, carried out by p38 MAPK, has been detected on GSK3β in brain^48^. Thus, it is possible that GSK3β following cold and β-adrenergic stimulation could also be inhibited by p38 MAPK activity, downstream of PKA, adding a second layer of regulation to GSK3 activity in brown adipocytes.

Small-molecule inhibitors of GSK3 are already being considered for the treatment of Alzheimer’s disease, bipolar disorder, certain cancers and type 2 diabetes^49,50^. Interestingly, studies with rodent models of metabolic disease have demonstrated that inhibiting GSK3 has beneficial effects on systemic metabolism, but also impact whole-body non-specific metabolic actions. GSK3 inhibitors were found to ameliorate diet-induced obesity, decrease adiposity and hepatic steatosis and improve glucose tolerance and lipid profiles^22–25^. These effects have mainly been accredited to improved insulin signaling, preservation of β-cell function and increased glycogen deposition in the liver. To our knowledge, the direct effect of GSK3 inhibitors on adipose tissue has not been studied. Here we show using cultured mouse brown adipocytes that inhibiting GSK3 can increase thermogenic gene expression, energy expenditure and adipokine secretion. The effects of inhibiting GSK3 in adipose tissue of mice and in human brown adipocytes remain to be shown.

To harvest the metabolic benefits of BAT, finding negative regulators of thermogenic activation is of importance, since these regulators might be druggable targets. Here, we propose that GSK3 is such a druggable target that has the potential to allow a release of the brake on BAT activity to impact whole-body energy metabolism.

## Methods

### Mice

Mice used in Figs 1 and 4 were single-caged 10 weeks old male C57BL/6JBomTac mice (Taconic), housed at room temperature and fed standard chow diet. For cold experiments, mice were kept at 4 °C or 30 °C (thermoneutrality) for 4 days. Animals were killed by cervical dislocation, and iBAT, aBAT, iWAT, eWAT, heart, skeletal muscle and liver were rapidly excised and snap frozen in liquid nitrogen and stored at −80 °C. Blood was collected by sub-mandibular puncture and serum was prepared. Mouse experiments were preapproved and conducted in accordance with the legislation of Danish authorities. The *AdipoQ*-caPKA mice used in Fig. 5 have been described^33^, and were maintained according to a protocol approved by the Institutional Animal Care and Use Committee of the University of Chicago.

### Cell culture

Brown pre-adipocytes immortalized with SV40 large T antigen were kindly provided by Dr. C. Ronald Kahn^51^. The cells were propagated in DMEM (Life Technologies) containing 10% fetal bovine serum (FBS) (Life Technologies). Two days post-confluence (designated day 0) differentiation was induced by addition of propagation medium supplemented with 1 μM dexamethasone (Sigma-Aldrich), 0.5 mM isobutyl-1-methylxanthine (IBMX) (Sigma-Aldrich), 5 μg/ml insulin (Roche) and 0.5 μM rosiglitazone (Cayman Chemical). At day 2, the medium was changed to medium containing 5 μg/ml insulin and 0.5 μM rosiglitazone. From day 4, cells were cultured in propagation medium and the cells were considered mature at day 8. The cells were kept at 37 °C in a humidified atmosphere with 5% CO_2_. 3T3-L1, C2C12 and hMADS cells were kindly provided by Dr. Karsten Kristiansen (3T3-L1, C2C12) and Dr. Christian Dani (hMADS)^52^. 3T3-L1 and hMADS cells were propagated and differentiated as described^53^. C2C12, HepG2 (ATCC) and Hepa1-6 (ATCC) cells were propagated in the same medium as the immortalized brown pre-adipocytes. Differentiation of C2C12 cells was induced by replacing the propagation medium with DMEM containing 2% horse serum from two days post-confluence (designated day 0 and considered as myoblasts). The cells were considered mature myocytes at day 2. HepG2 and Hepa1-6 cells were harvested at 80% confluence. Primary CD-1 mouse hepatocytes were obtained from Life Technologies (MSCP10) and were propagated according to the manufacturer’s instructions. All media contained 62.5 μg/ml penicillin and 100 μg/ml streptomycin (Sigma-Aldrich).

### Isolation and culture of primary brown adipocytes

Isolation and culture of primary brown pre-adipocytes from male 3–4 weeks old NMRI mice (Taconic) were done as described^53^, except that the cell culture medium was changed at days 1, 4, 6 and 8 after isolation.

### Gene expression

Isolation of total RNA, reverse transcription and RT-qPCR was done as described^54^, except that the SensiFAST SYBR Lo-ROX Kit was used. Primers used were: *Tbp*, fwd-ACCCTTCACCAATGACTCCTATG, rev-ATGATGACTGCAGCAAATCGC; *Ucp1*, fwd-AGCCGGCTTAATGACTGGAG, rev-TCTG TAGGCTGCCCAATGAAC; *Fgf21*, fwd-AGATGGAGCTCTCTATGGATCG, rev-GGGCTTCAGACTGGTACACAT; *Fabp4*, fwd-TGGAAGCTTGTCTCCAGTGA, rev-AATCCCCATTTACGCTGATG; *AdipoQ*, fwd-AACTTGTGCAGGTTGGATGGC, rev-TTCTCTCCCTTCTCTCCAGGA; *Cebpa*, fwd-TGGACAAGAACAGCAACGAG, rev-TCACTGGTCAACTCCAGCAC; *Dio2*, fwd-CAGTGTGGTGCACGTCTCCAATC, rev-TGAACCAAAGTTGACCACCAG; *Ppargc1a*, fwd-AGCCGTGACCACTGACAACGAG, rev-GCTGCATGGTTCTGAGTGCTAAG.

### FGF21 secretion measurements

FGF21 levels in plasma and cell culture medium were determined with the Mouse/Rat FGF-21 Quantikine ELISA Kit (R&D Systems) according to the instructions of the manufacturer.

### Kinase inhibitors and activators

The Tocriscreen Kinase Inhibitor Toolbox containing 80 kinase inhibitors was supplemented with 10 additional inhibitors (Tocris Bioscience)^28^. SB415286, SB216763, BIO, H89 and SB202190 were obtained from Tocris Bioscience. 6-MB-cAMP was from Biolog. All compounds were dissolved in DMSO, except for 6-MB-cAMP which was dissolved in water.

### Immunoblotting

Preparation of whole-cell extracts and immunoblotting were carried out essentially as described^55^. Briefly, protein lysates were separated using NuPage 4–12% Bis-Tris gradient gels (Life Technologies) and transferred by semi-dry blotting onto polyvinylidene difluoride membrane (GE Healthcare). Equal loading was confirmed by Amido Black staining (Sigma-Aldrich). All washing and incubation steps were carried out with Tris-buffered saline containing 0.1% Tween-20 and 5% non-fat dry milk or BSA. Primary antibodies used were: CREB (#9192), p-CREB (Ser133) (#9198), GSK3α (#4337), p-GSK3α (Ser21) (#9316), GSK3β (#12456), p-GSK3β (Ser9) (#5558), p38 MAPK (#9212), p-p38 MAPK (Thr180/Tyr182) (#9211), MKK3 (#8535), MKK6 (#8550), p-MKK3/6 (Ser189/Ser207) (#12280), ATF2 (#9226), p-ATF2 (Thr71) (#5112), HSL (#4107), p-HSL (Ser660) (#4126), Phospho-(Ser/Thr) PKA substrate (#9621) (all from Cell Signaling Technology), TFIIB (#sc-225) (Santa Cruz Biotechnology), Vinculin (#V9264) (Sigma-Aldrich), UCP1 (#10983) (Abcam) and HA (#11583816001) (Roche). Secondary antibodies were horseradish peroxidase-conjugated anti-rabbit or anti-mouse (DAKO). EZ-ECL Enhanced Chemiluminescence Detection Kit for HRP (Biological Industries) was used for detection.

### Retroviral overexpression

Human pcDNA3-HA-GSK3β-S9A and human pcDNA3-HA-GSK3β-K85A were a gift from Jim Woodgett (Addgene plasmid number #14755 and #14754). GSK3β fragments were inserted into the *Eco*RI/*Apa*I site of pMSCVneo link3^54^ to create pMSCVneo-HA-GSK3β-S9A and pMSCVneo-HA-GSK3β-K85A. Phoenix-Eco cells were transfected with retroviral vectors at 50–60% confluence using Fugene HD Transfection Reagent (Promega). 48 and 72h after transfection, the virus-containing supernatant was harvested and filtered. Subconfluent immortalized brown preadipocytes were transduced with the virus-containing supernatant diluted with DMEM containing 10% FBS and supplemented with 4.5 μg/ml polybrene (Sigma-Aldrich). Transduced cells were selected with 400 μg/ml G418 (Sigma-Aldrich).

### Reverse siRNA transfection

Reverse siRNA transfections were performed as described^53^. siRNAs used were GSK3α (SASI_Mm01_00126759) and GSK3β (SASI_Mm01_00141911) (Sigma-Aldrich). The MISSION® siRNA Universal Negative Control #1 (Sigma-Aldrich, SIC001) was used as control siRNA.

### Oxygen consumption measurements

Real-time measurements of oxygen consumption rate (OCR) were performed using a Seahorse XF96 Extracellular Flux Analyzer (Agilent Technologies). Mature primary brown adipocytes were replated from 6-well plates at day 8 after isolation and reverse transfected in 96-well XF Cell Culture Microplates (Agilent Technologies) as previously described^53^. Cells were kept in growth medium until the day of the experiment, i.e. 4 days after transfection. The cell culture medium was changed 1 h before the first measurement to DMEM (without serum) supplemented with 5 mM glucose and adjusted to pH 7.4. OCR was measured under basal conditions and following isoproterenol injection.

### Glycerol release

Glycerol release into the cell culture medium was measured with the Adipolysis Assay Kit (Cayman Chemical) following the instructions of the manufacturer.

### Statistics

All statistical tests of gene expression were performed on log-transformed data. Unpaired two-tailed Student’s t-test was used for single comparisons. Two-way ANOVA with repeated measures and Tukey’s post hoc test was used for multiple comparisons.

## Electronic supplementary material

Supplementary Information
