# Supplementary material for: GSK3 is a negative regulator of the thermogenic program in brown adipocytes
Source: Sci Rep. 2018 Feb 22;8:3469. doi: 10.1038/s41598-018-21795-y (PMC5823915; doi:10.1038/s41598-018-21795-y)
Supplement: Supplementary file 1 — Supplementary Information [file 41598_2018_21795_MOESM1_ESM.pdf]

GSK3 is a negative regulator of the thermogenic program in brown adipocytes

Lasse K. Markussen, Sally Winther, Barton Wicksteed & Jacob B. Hansen

Supplementary Information Files

Supplementary Figure 1: GSK3 is an adipocyte-selective Fgf21 regulator.

Supplementary Figure 2: Full-length immunoblots.

Supplementary Table 1: Inhibitors used for the kinase inhibitor screens.

Supplementary Table 2: Quantification of immunoblots.

Supplementary Figure 1

a

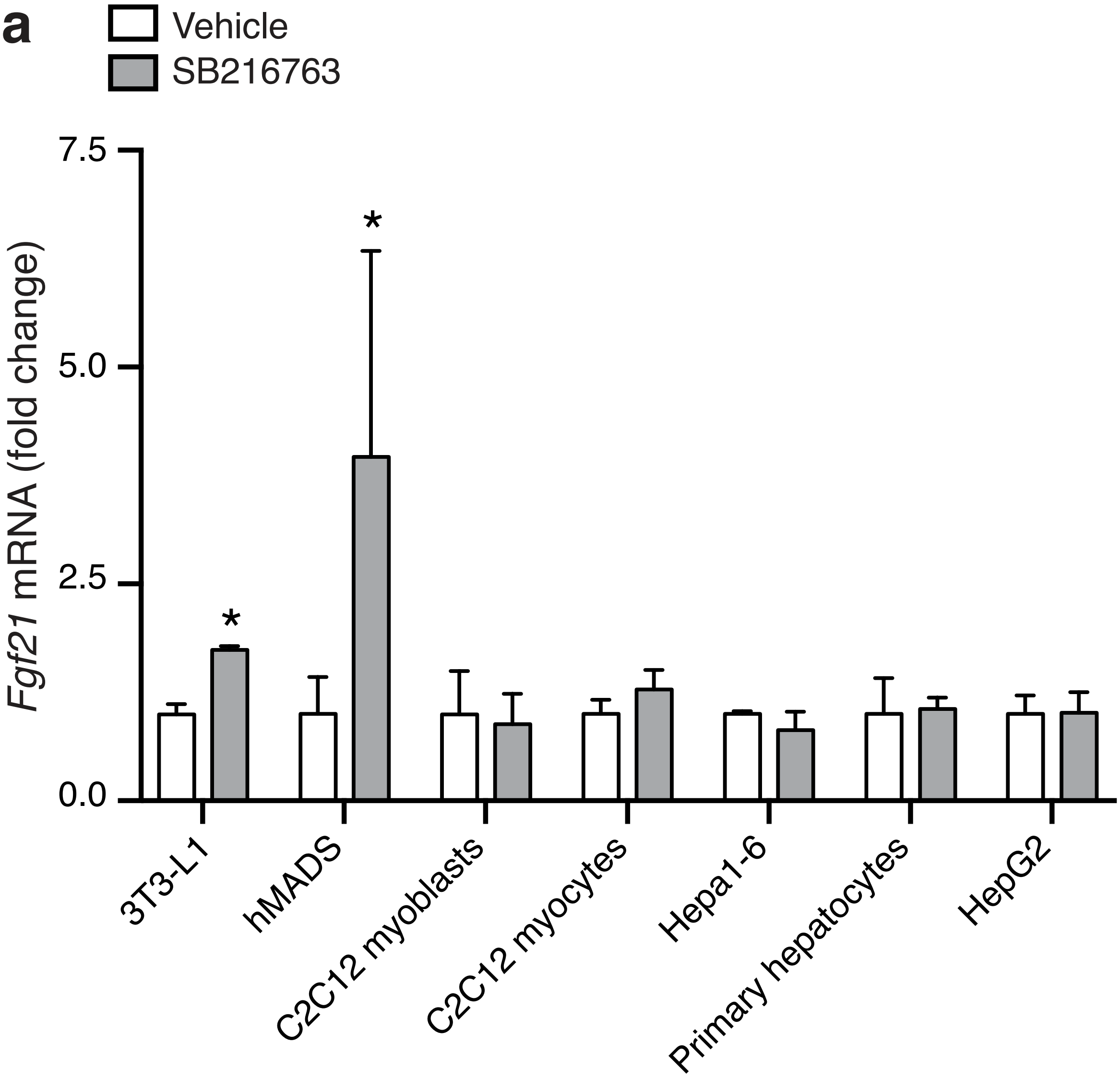

Supplementary Figure 2: Full-length immunoblots.

Fig. 2d:

HA-tag GSK3b TFIIB

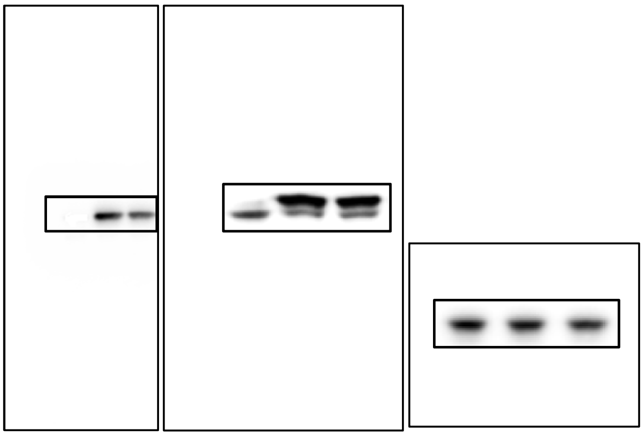

Fig. 3b:

UCP1 TFIIB

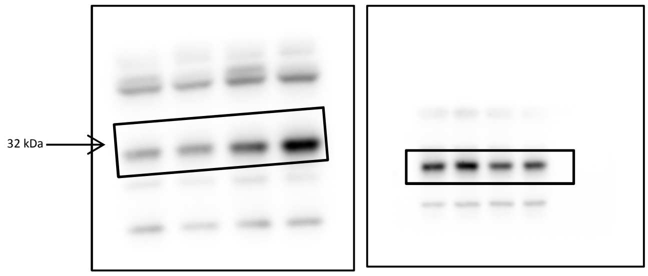

Fig. 3d:

TFIIB

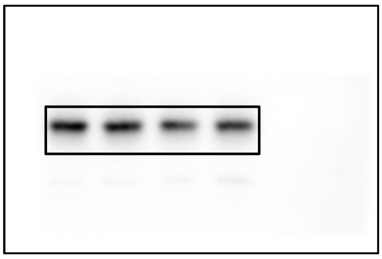

GSK3b

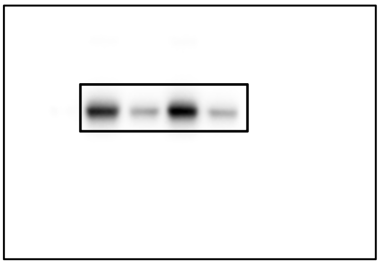

GSK3a

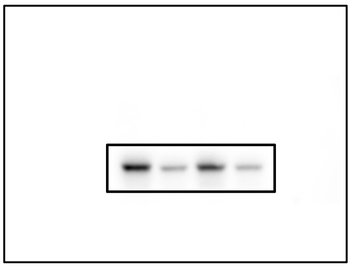

Fig. 4a:

GSK3a

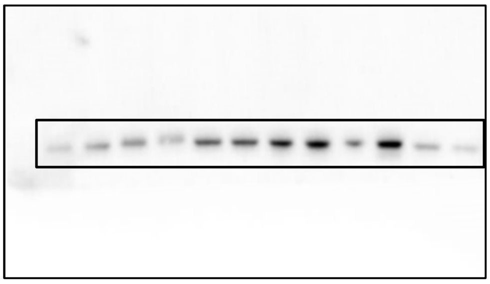

GSK3b

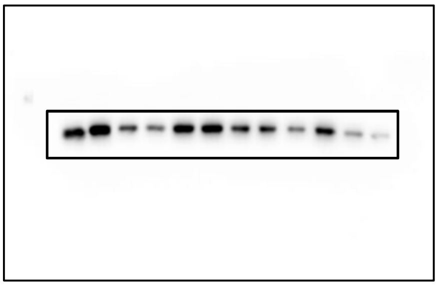

Vinculin

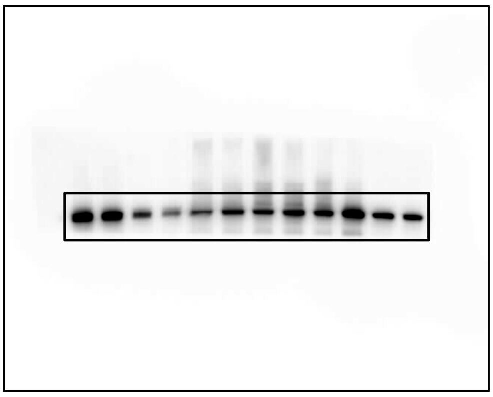

Fig. 4b:

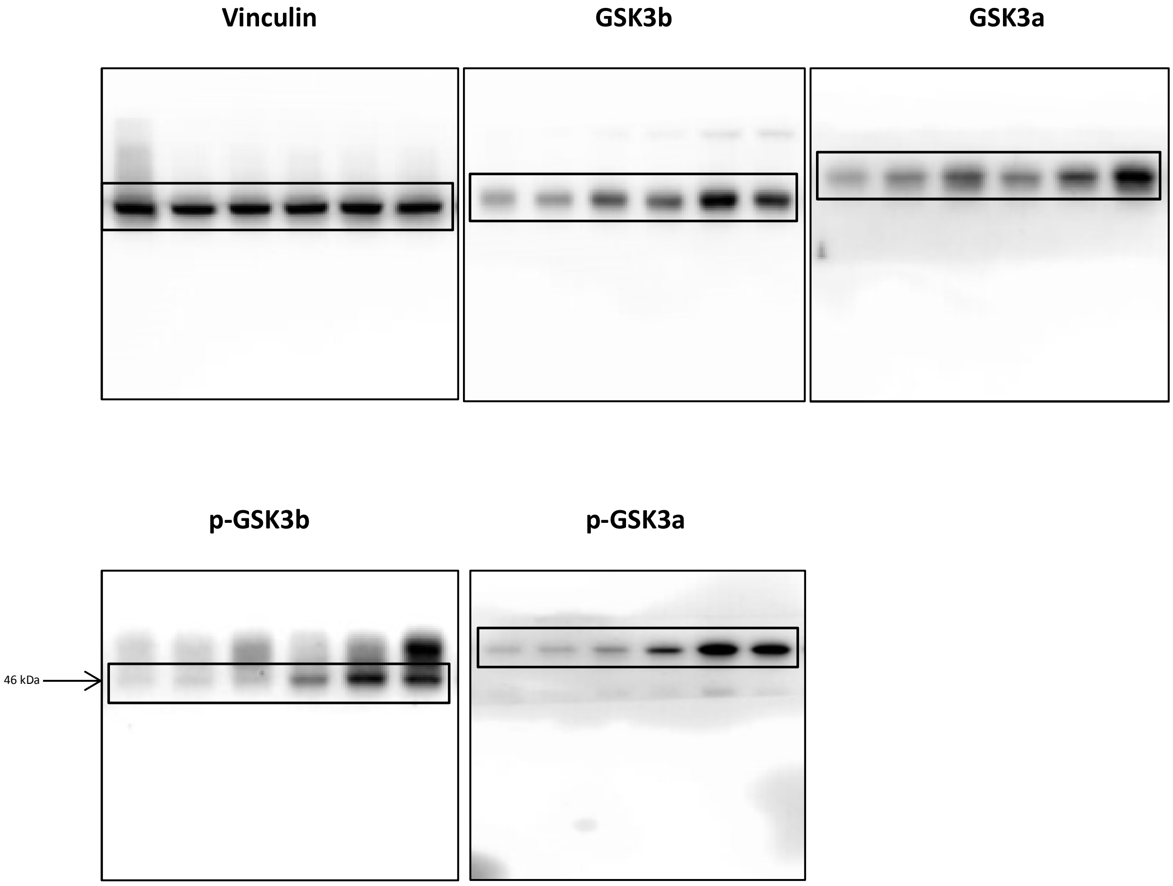

Fig. 4c:

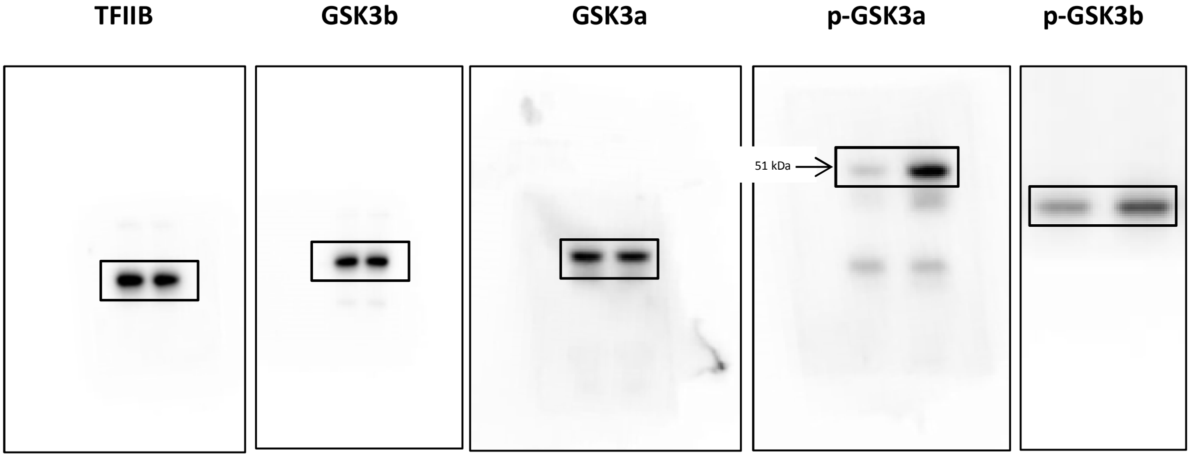
TFIIB
GSK3b
GSK3a
p-GSK3a
p-GSK3b
51 kDa

Fig. 5b:

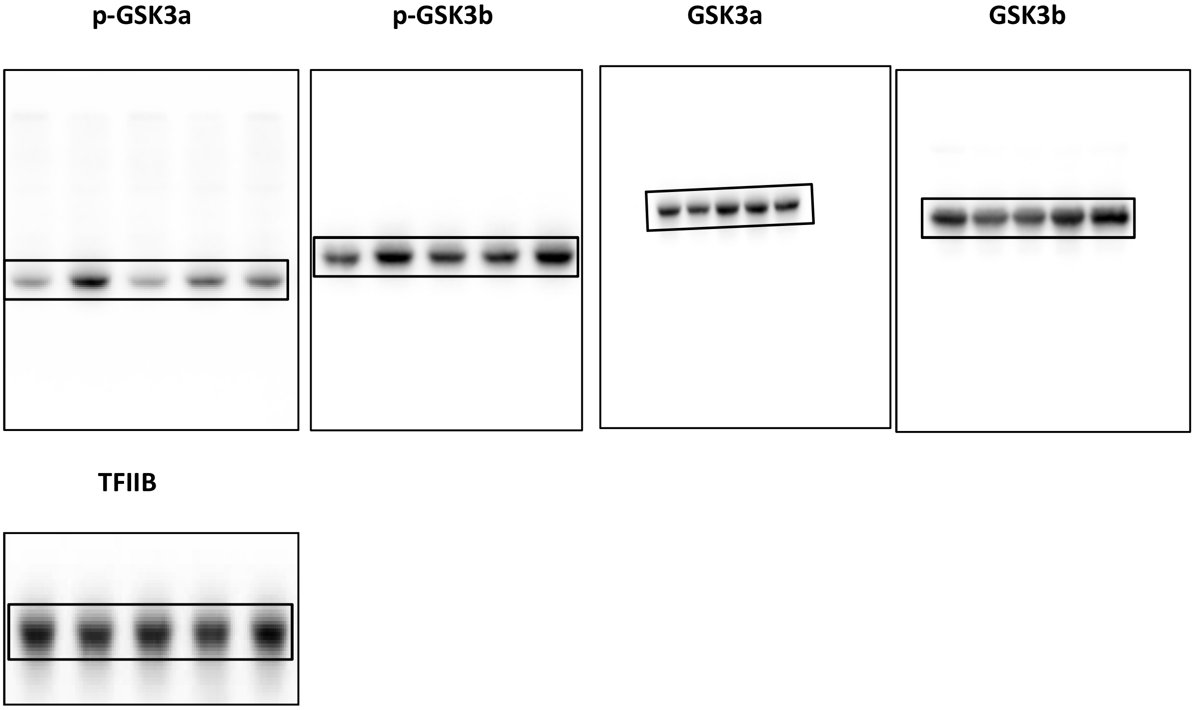
p-GSK3a
p-GSK3b
GSK3a
GSK3b
TFIIB

Fig. 5c:

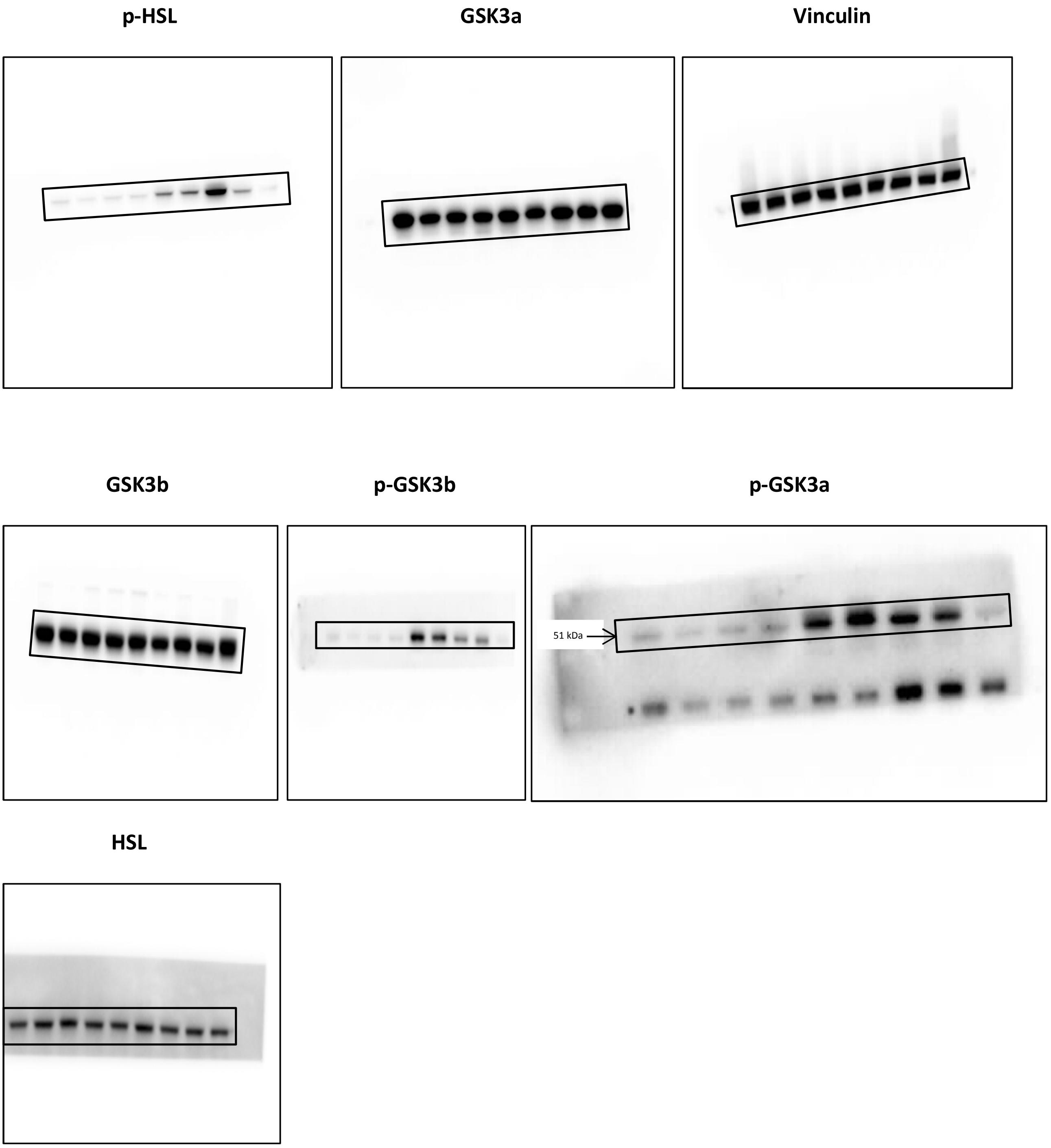

Fig. 5e:

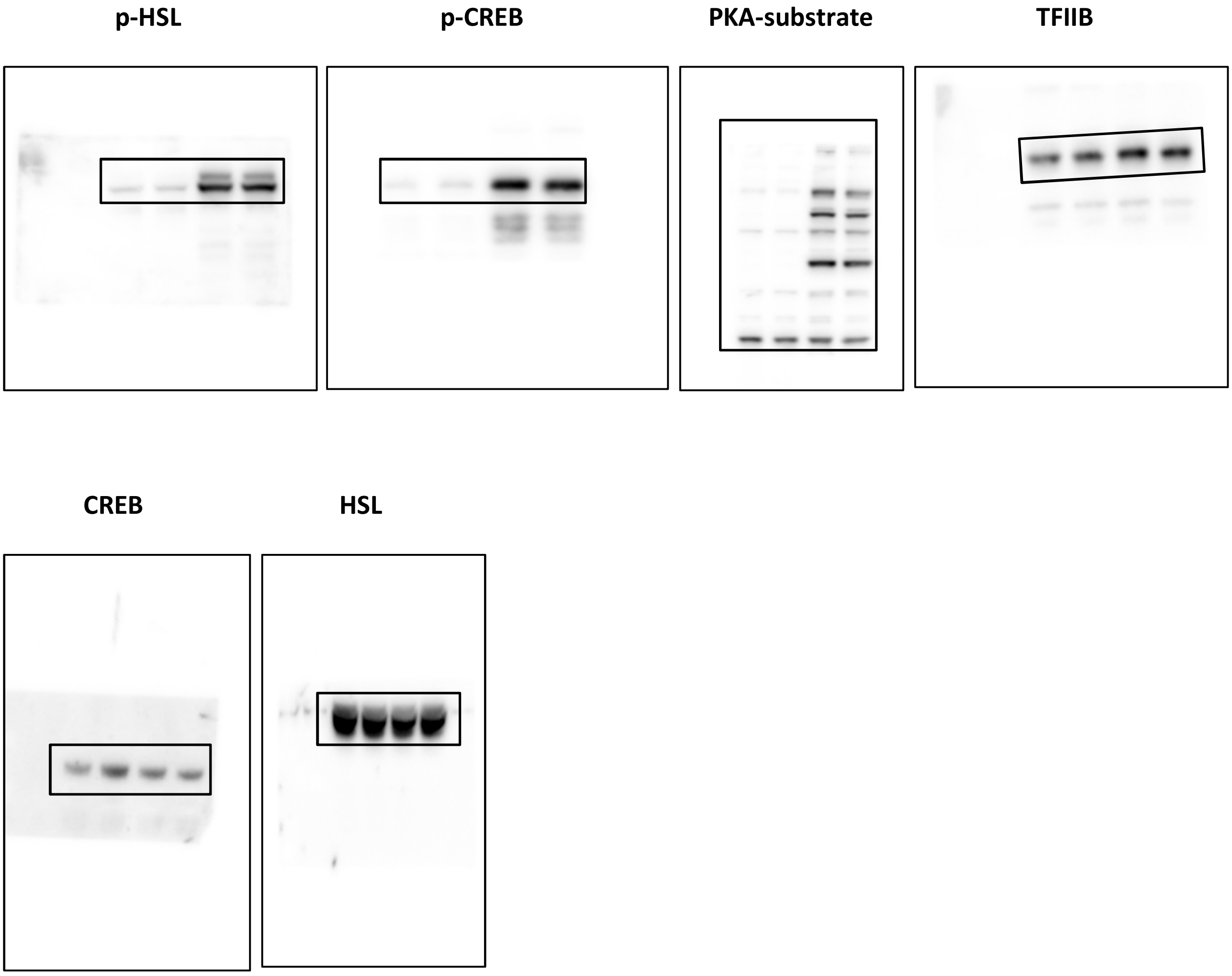

Fig. 6a:

p-ATF2

Above = p-MKK3/6

Below = TFIIB

P-p38

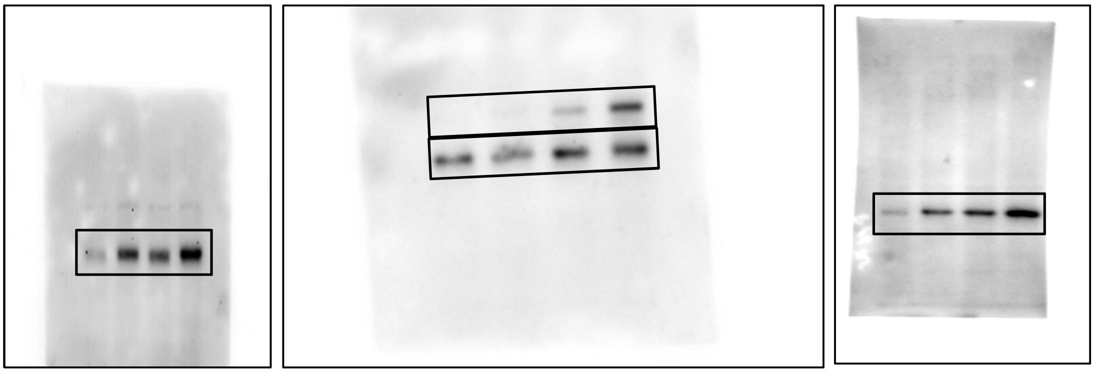

P38

p-AKT

AKT

MKK6

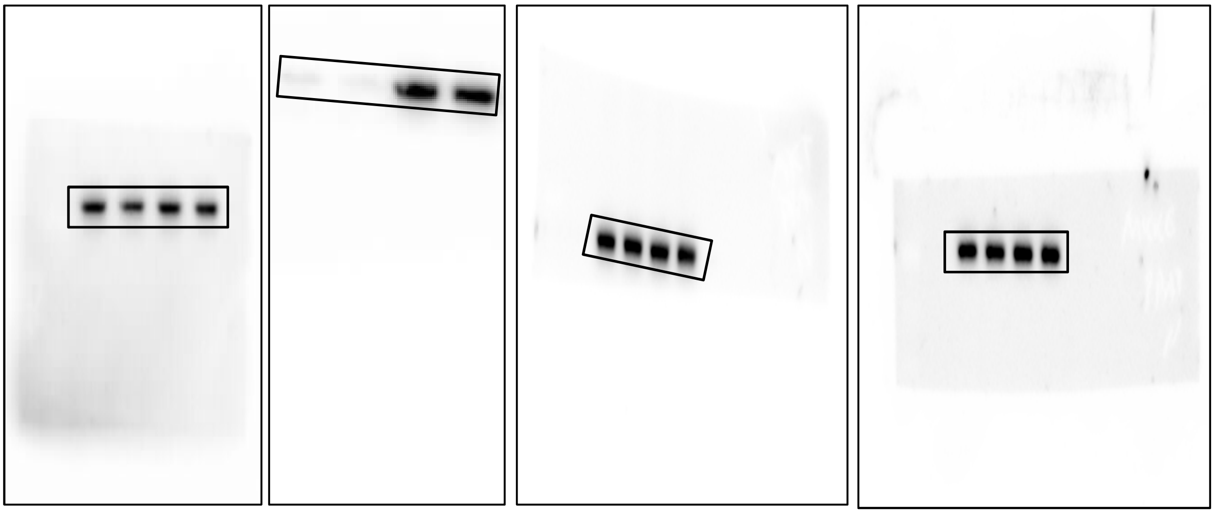

MKK3

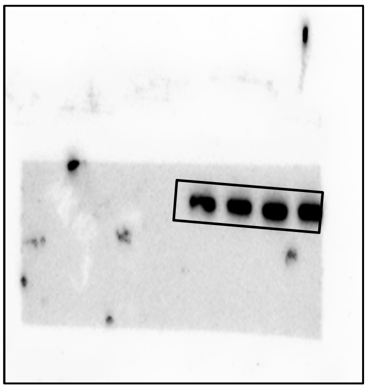

ATF2

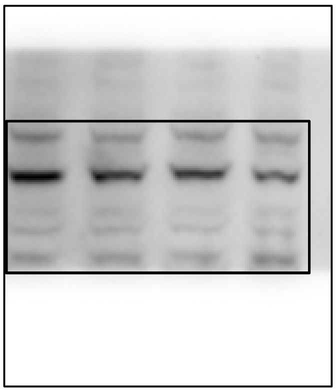

Fig. 6b:

p-p38

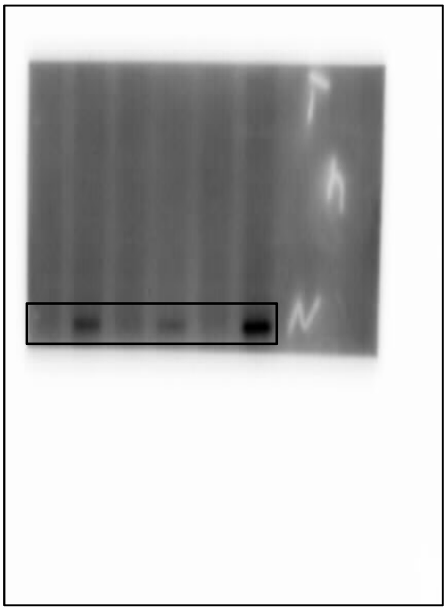

p38

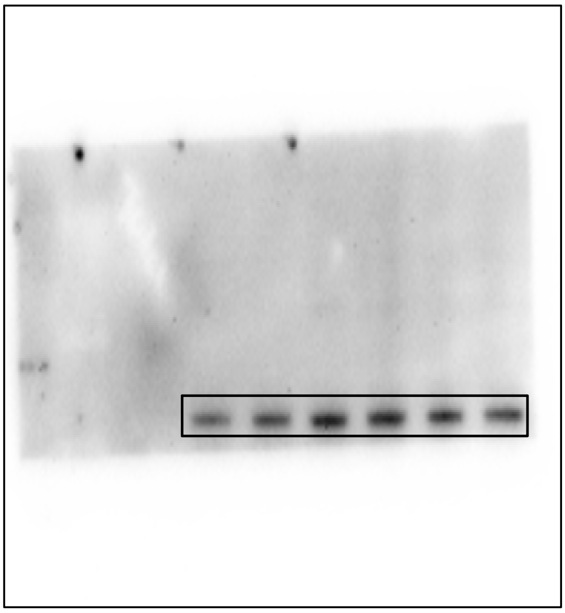

Supplementary Table 1

No.	Inhibitor	Primary target
1	(DMSO)	(Unstimulated control)
2	K252a	Broad spectrum
3	Ro 31-8220 mesylate	Broad spectrum
4	ER 27319 maleate	SYK
5	Olomoucine	cdk
6	PD 407824	Chk1
7	Ryuvidine	cdk
8	IMD 0354	IKK
9	GF 109203X	PKC
10	SB 218078	Chk1
11	Ki 8751	VEGFR
12	H 89 dihydrochloride	PKA
13	API-2	PKB
14	LFM-A13	BTK
15	SB 239063	p38
16	SB 202190	p38
17	Dorsomorphin dihydrochloride	AMPK
18	Aminopurvalanol A	cdk
19	ZM 336372	Raf
20	ZM 449829	JAK3
21	SB 203580 hydrochloride	p38
22	Fasudil hydrochloride	ROCK
23	(-)-Terreic acid	BTK
24	10-DEBC hydrochloride	PKB
25	PI 828	PI3K
26	NSC 693868	cdk
27	Y-27632 dihydrochloride	ROCK
28	GW 583340 dihydrochloride	EGFR
29	PD 198306	MEK
30	PHA 665752	cMET
31	Ro 08-2750	TrkA
32	EO 1428	p38
33	PP 1	Src
34	GW 441756	TrkA
35	Rapamycin	mTOR
36	AG 490	EGFR
37	TBB	CK2
38	BI 78D3	JNK
39	ZM 447439	Aurora
40	BIO	GSK-3
41	GW 5074	Raf
42	CGK 733	ATR/ATM
43	Iressa	EGFR
44	ZM 39923 hydrochloride	JAK3
45	SL 327	MEK
46	KU 55933	ATM
47	TCS 359	FLT3
48	SU 4312	VEGFR
49	1-Naphthyl PP1	Src
50	SU 5416	VEGFR
51	KH7	Adenylyl cyclase
52	SC 514	IKK
53	ZM 323881 hydrochloride	VEGFR
54	ML 9 hydrochloride	MLCK
55	GSK 650394	SGK
56	1,2,3,4,5,6-Hexabromocyclohexane	JAK2
57	HA 1100 hydrochloride	ROCK
58	Arcyriaflavin A	cdk
59	BIBX 1382 dihydrochloride	EGFR
60	PD 98059	MEK
61	Purvalanol B	cdk
62	SD 208	TGFbR1
63	FPA 124	PKB
64	KT 5720	PKA
65	IKK 16	IKK
66	PQ 401	IGF-R1
67	D 4476	CK1
68	NU 7026	DNA-PK
69	KT 5823	PKG
70	LY 364947	TGFbR1
71	Compound 401	DNA-PK
72	GW 843682X	PLK
73	LY 294002 hydrochloride	PI3K
74	PP 2	Src
75	Genistein	EGFR
76	CGP 57380	Mnk1
77	Purvalanol A	cdk
78	KN-62	CamKII
79	ZM 306416 hydrochloride	VEGFR
80	CGP 53353	PKC
81	TPCA-1	IKK
82	SQ 22536	Adenylyl cyclase
83	U0126	MEK
84	SB 216763	GSK-3
85	SP 600125	JNK
86	KU0063794	mTOR
87	SB 415286	GSK-3
88	Arctigenin	MEK
89	SB 431542	TGFbR1
90	NH 125	CamKIII
91	STO-609 acetate	CaMKK/AMPK

Supplementary Table 2

**Fig. 2d**	GSK3β/TFIIB	HA/TFIIB
Vector	1.00	ND
CA	5.33	1.00
KD	5.44	0.48

**Fig. 3b**	UCP1/TFIIB
Vehicle	1.00
Vehicle + SB216763	1.00
ISO	2.78
ISO + SB216763	4.78

**Fig. 3c**	GSK3α/TFIIB	GSK3β/TFIIB
Vehicle	1.00	1.00
Vehicle + siGSK3	0.32	0.33
ISO	1.04	1.40
ISO + siGSK3	0.33	0.34

**Fig. 4a**	GSK3α/Vinculin	GSK3β/Vinculin
Heart	0.44	1.41
SKM	2.72	3.75
iBAT	1.61	2.12
iWAT	1.39	0.91
eWAT	0.86	0.96
Liver	0.49	0.47

**Fig. 4b**	p-GSK3α/GSK3α	p-GSK3β/GSK3β
RT	1.00	1.00
Cold	3.95	2.72

**Fig. 4c**	p-GSK3α/GSK3α	p-GSK3β/GSK3β
Vehicle	1.00	1.00
ISO	7.43	2.52

**Fig. 5b**	p-GSK3α/GSK3α	p-GSK3β/GSK3β
Vehicle	1.00	1.00
ISO	2.55	1.92
H89	0.69	1.29
ISO + H89	1.20	1.15
6-MB	1.31	1.56

**Fig. 5c**	p-HSL/HSL	p-GSK3α/GSK3α	p-GSK3β/GSK3β
Wild-type	1.00	1.00	1.00
AdipoQ-caPKA	16.69	3.07	3.19

**Fig. 5e**	p-HSL/HSL	p-CREB/CREB
Vehicle	1.00	1.00
SB216763	1.68	0.81
ISO	13.13	13.62
ISO + SB216763	11.60	16.23

**Fig. 6a**	p-AKT/ AKT	p-MKK3/6 / MKK6	p-p38/ p38	p-ATF2/ ATF2
Vehicle	1.00	ND	1.00	1.00
SB216763	0.95	ND	1.90	5.17
ISO	16.38	1.00	3.41	6.42
ISO + SB216763	17.52	2.22	7.29	23.90

**Fig. 6b**	p-p38/p38
Vector	1.00
Vector + ISO	7.57
CA	1.27
CA + ISO	1.65
KD	0.08
KD + ISO	28.26
